# Transcriptomic analysis of the mouse retina after acute and chronic normobaric and hypobaric hypoxia

**DOI:** 10.1038/s41598-021-96150-9

**Published:** 2021-08-17

**Authors:** L. J. A. Ebner, M. Samardzija, F. Storti, V. Todorova, D. Karademir, J. Behr, F. Simpson, M. Thiersch, C. Grimm

**Affiliations:** 1 Laboratory for Retinal Cell Biology, Department of Ophthalmology, University Hospital Zurich, University of Zurich, Wagistrasse 14, Schlieren, 8952 Zurich, Switzerland; 2 Zurich Center for Integrative Human Physiology, University of Zurich, Winterthurerstrasse 190, 8057 Zurich, Switzerland; 3 Neuroscience Center Zurich (ZNZ), University of Zurich, Winterthurerstrasse 190, 8057 Zurich, Switzerland; 4 Institute of Veterinary Physiology, Vetsuisse Faculty, University of Zurich, Winterthurerstrasse 260, 8057 Zurich, Switzerland

**Keywords:** Retina, RNA sequencing

## Abstract

Oxygen delivery to the retinal pigment epithelium and the outer retina is essential for metabolism, function, and survival of photoreceptors. Chronically reduced oxygen supply leads to retinal pathologies in patients and causes age-dependent retinal degeneration in mice. Hypoxia can result from decreased levels of inspired oxygen (normobaric hypoxia) or reduced barometric pressure (hypobaric hypoxia). Since the response of retinal cells to chronic normobaric or hypobaric hypoxia is mostly unknown, we examined the effect of six hypoxic conditions on the retinal transcriptome and photoreceptor morphology. Mice were exposed to short- and long-term normobaric hypoxia at 400 m or hypobaric hypoxia at 3450 m above sea level. Longitudinal studies over 11 weeks in normobaric hypoxia revealed four classes of genes that adapted differentially to the hypoxic condition. Seventeen genes were specifically regulated in hypobaric hypoxia and may affect the structural integrity of the retina, resulting in the shortening of photoreceptor segment length detected in various hypoxic groups. This study shows that retinal cells have the capacity to adapt to long-term hypoxia and that consequences of hypobaric hypoxia differ from those of normobaric hypoxia. Our datasets can be used as references to validate and compare retinal disease models associated with hypoxia.

## Introduction

The retina is one of the most metabolically active tissues in our body^1^ and consumes large amounts of oxygen to maintain its function. Diseases affecting the choriocapillaris and/or the central retinal artery lead to pathological changes including reduced vessel diameter, as well as decreased velocity and oxygen-carrying capacity of red blood cells, resulting in reduced tissue oxygenation (hypoxia). Retinal hypoxia occurs in various retinal diseases such as diabetic retinopathy^2^, ischemic central retinal vein thrombosis^3^, and neovascular age-related macular degeneration (nAMD)^4^. Recent evidence suggests that mild, but chronic hypoxic conditions, that may develop due to reduced choroidal blood flow and volume^5–7^, choroidal ischemia^8^, and accumulation of drusen may also be important for the development and progression of the non-exudative form of AMD^8^. The involvement of hypoxia in disease is further supported by recent computational models showing a strong correlation between hypoxia and retinal thinning during AMD development^9^.

In contrast to potential long-term adaptation of people living in the highlands (reviewed in^10^), ascent to high altitude by a non-acclimatized person can cause acute mountain sickness^11^, memory loss, high-altitude cerebral edema^12^, and high-altitude retinopathy that belongs to the group of hypoxia-regulated diseases^13^. Several studies point to the physiological differences in the adaptation to normobaric versus hypobaric hypoxia. Hypobaric hypoxia leads to greater hypoxemia, hypocapnia, and blood alkalosis, but lower arterial oxygen saturation and ventilation^14,15^. Although several morphological and functional changes have been reported in the hypobaric hypoxic eye, including optic disc swelling^16^, vitreous and retinal hemorrhages, as well as retinal and choroidal blood flow alterations^17–19^, the molecular processes underlying these changes remain mostly unclear.

Since reduced tissue oxygenation and the resulting pathological processes are implicated in many retinal diseases, more insights into the cellular response to acute and chronic hypoxia are necessary to better understand disease development and progression. In this study, we investigated the transcriptomic differences in retinas of six groups of mice that were exposed to either normobaric or hypobaric hypoxia for various durations. Additionally, the impact of different hypoxic conditions on photoreceptor segment length was evaluated.

## Results

### Physiological parameters of mice exposed to hypoxia

To gain understanding of the response of retinal cells to reduced tissue oxygenation, we analyzed the consequences of acute and chronic normobaric and hypobaric hypoxia for the retinal transcriptome in seven groups of mice. Four experimental groups (denoted ‘ZH’) were set to analyze transcriptomic changes during normobaric hypoxia of varying levels (7% or 14% O_2_) and duration (6 h, 48 h, and 11 weeks), and two groups were evaluated in hypobaric hypoxia (denoted 'JFJ') after exposure to reduced barometric pressure (at 3450 m above sea level (masl)) for 48 h or 7 weeks (Table 1). An additional group served as normoxic control. A normobaric concentration of 14% inspired O_2_ was chosen because it corresponds to the hypobaric condition at the high-altitude research station at 3450 masl. This allowed the direct comparison of the consequences induced by normobaric and hypobaric hypoxia.

**Table 1:** Experimental groups.

Nomenclature	O_2_ conc. (%)	Duration (weeks, h)	Location	Altitude (masl)	Barometric pressure (kPa)	Number of mice for RNA seq	Age^#^ (weeks)
Normoxia	21	11 w	ZH	408	97	6	9.5
07-06h-ZH	7	06 h	ZH	408	97	6	10
14-06h-ZH	14	06 h	ZH	408	97	6	9–11
14-48h-ZH	14	48 h	ZH	408	97	6	9–11
14-11w-ZH	14	11 w	ZH	408	97	6	9.5
14-48h-JFJ	≈ 14	48 h	JFJ	3450	65	6	9
14-07w-JFJ	≈ 14	07 w	JFJ	3450	65	6	9

masl: meter above sea level; ZH: Zürich; JFJ: Jungfraujoch high-altitude research station; ^#^age at the beginning of the experiment.

While hypoxic preconditioning by short-term exposure to 7% O_2_ was previously shown to be protective against light damage^20^, a computational model suggests that chronically low O_2_ concentrations below 10 mmHg may cause retinal thinning^9^. Thus, investigating the retinal response to 7% O_2_ may help to understand the events that either lead to protection or degeneration.

The body responds to chronic hypoxia by stimulating erythropoiesis to increase hematocrit and hemoglobin levels, which ultimately improves the oxygen transport capacity of the blood^21,22^. Therefore, we determined hemoglobin levels and hematocrit values in mice subjected to 14% normobaric or hypobaric hypoxia for two days or longer (48 h–11 w). Since in humans hemoglobin levels and hematocrit are usually not significantly influenced within the first 48 h of mild hypoxia^23^, the 6 h time-point was excluded from this analysis. Both hemoglobin and hematocrit levels were elevated in the two long-term hypoxia groups (14-11w-ZH, 14-07w-JFJ) compared to normoxic controls, confirming physiological response to sustained reduced oxygen levels. The short-term (48 h) normobaric hypoxic mice (14-48 h-ZH), however, had hemoglobin and hematocrit values comparable to the normoxic controls. Interestingly, mice exposed to hypobaric hypoxia for 48 h (14-48 h-JFJ) had lower hemoglobin levels than mice in normoxia, and a tendency for reduced hematocrit that did not reach statistical significance (Fig. S1; Table S1).

To test whether hypoxic exposure affects the retina in general, which could influence the transcriptomic analysis, retinal sections of all experimental groups were stained for glial fibrillary acidic protein (GFAP) to test for general tissue stress and activation of Müller glia cells, allograft inflammation factor 1 (AIF1 or IBA1) to investigate potential microglia activation, and Isolectin b4 to address potential effects on the retinal vasculature. The corresponding immunostainings were indistinguishable from normoxic controls (Fig. S2). This suggests that even prolonged hypoxic exposure had no impact on tissue stress or the retinal vasculature.

### Differentially expressed genes in the retina of mice exposed to hypoxia

Preceding note: to increase the readability of the text, we define only the abbreviations of those genes that are specially addressed in the discussion. For the definition of all other gene names, please refer to the respective Ensembl gene webpage (https://www.ensembl.org).

To investigate retinal gene expression in response to acute and chronic normobaric and hypobaric hypoxia, we analyzed the retinal transcriptome of mice from all 7 groups. Since retinas were isolated under different conditions at the research lab in Zurich and the Jungfraujoch (JFJ) research station, we first tested the RNA-Seq data for expression of marker genes specific for the various retinal cell types^24^ to validate the cellular composition of the tissue in each sample. The heat map of normalized counts showed that all samples had comparable expression levels of the marker genes, suggesting that the individual samples had a similar content of retinal cells within and across groups (Fig. S3).

To further validate the RNA-Seq data, we verified the expression of 6 hypoxia-regulated genes, namely *Adm, Bnip3, Egln1, Slc2a1, Pdk1* and *Vegfa* by real-time PCR (Table S2). qPCR data compared best to RNA-Seq data for genes with a fold change above 2, therefore we used a fold change threshold of 2 (log_2_FC of ≥ ± 1) for deep RNA-Seq data analyses. Prior to the analysis, we defined a list of genes (Table S3) that likely originated from residual retinal pigment epithelium (RPE)/lens/iris tissue contamination in the samples. This list included hemoglobins, as well as known RPE markers (e.g. *Rpe65*, *Mlana,* and *Pmel*) and various types of crystallins. Although the different levels of crystallin mRNAs were not necessarily due to tissue contamination, as some crystallins are also expressed in the retina^25^, they were nevertheless excluded from further analysis because it was not possible to discriminate between contamination from adjacent tissue and retina-specific signals.

**Figure 1:**
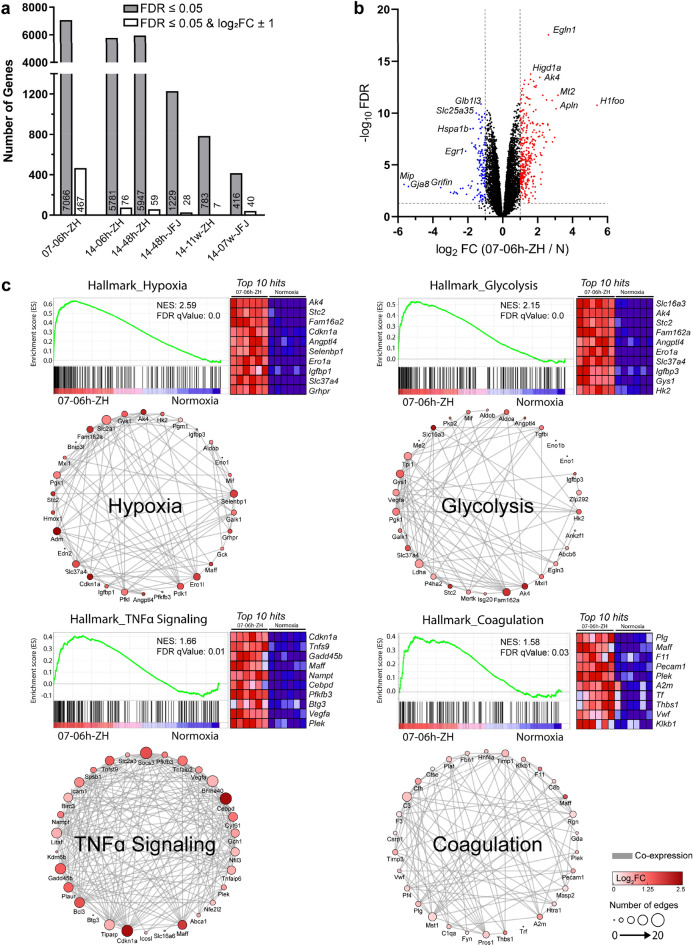
Differentially expressed genes in the retina after hypoxic exposure. (**a**) Number of differentially expressed genes identified for each hypoxic condition. Shown are genes with an FDR ≤ 0.05 (grey bars) and genes that were additionally filtered for an FC ≥  ± 2 (white bars) compared to normoxic controls. Nomenclature of groups is defined according to Table 1. (**b**) Volcano plot representing differentially expressed genes in the 7% acute normobaric hypoxia (07-06 h-ZH) group. Up-regulated genes are shown in red and down-regulated genes in blue. Plot shows − log_10_ FDR versus log_2_ FC of each gene detected in acute hypoxia relative to normoxia (N). Threshold limit lines are set at an FDR of 0.05 and log_2_FC of ± 1. (**c**) Gene set enrichment analysis (GSEA) of 07-06 h-ZH (n = 6) compared with normoxia (n = 6) with MSigDB hallmark datasets. Heatmaps represent the top 10 upregulated genes in hypoxia for each hallmark set. Co-expression networks of top 30 enriched genes of each set. Nomenclature: ‘Oxygen percentage—duration of hypoxia—location of experiment’ is given in the name of each experimental group. ZH denotes normobaric and JFJ hypobaric hypoxic groups. N: normoxia; FC: fold change; FDRq: false discovery rate; NES: normalized enrichment score.

Pairwise comparisons of each hypoxic group to normoxia were performed and differentially expressed (DE; FDR ≤ 0.05 and log_2_FC ≥ ± 1) genes identified. The highest number of DE genes was found in the group exposed to the lowest oxygen level for 7 h (07-06 h-ZH). A total of 467 differentially regulated genes (338 up- and 129 down-regulated; log_2_FC of ≥ ± 1) were identified among the 7066 genes with an FDR ≤ 0.05 cutoff (Fig. 1a,b; Supplementary Dataset 1). Of these genes, 442 were unique to the acute 07-06 h-ZH normobaric group (for the complete list of these genes see Supplementary Dataset 2), whereas the 25 remaining genes were identified in at least one other group. The number of DE genes was lower when mice were exposed to milder (14% O_2_) acute normobaric hypoxia (14-06 h-ZH) and declined further when mice were exposed for an extended time period, with the lowest numbers of DE genes identified in the two groups of chronic hypoxia (Fig. 1a). The chronic normobaric group (14-11w-ZH) had only seven genes differentially regulated. While one gene was up-regulated (*2610528A11Rik*), six genes were down-regulated in this group (Supplementary Dataset 1), of which three (*Fosl2, Nr4a1,* and *Nr4a3*) are involved in the regulation of gene expression.

Gene set enrichment analysis (GSEA) was used to identify changes in the 07-06 h-ZH group versus normoxia and revealed a strong positive correlation in genes associated with hypoxia (MiSgDB Hallmark gene set, normalized enrichment score (NES): 2.59), glycolysis (NES: 2.15), TNFα-Signaling via NFκB (NES: 1.66) and coagulation (NES: 1.59). Co-expression networks of the top 30 genes of each category identified the most interconnected genes (hubs) (Fig. 1c, Supplementary Dataset 3). For hypoxia especially HIF1α target genes like *Selenbp1*, *Adm*, *Slc2a1* (*Glut1*) and *Fam162α* are highly interconnected. The glycolysis network represents gene hubs strongly involved in glucose metabolism, like *Gys1*, *Pgk1,* and *Ldha*. Larger interconnections were found in the TNFα signaling, where *Cebpd* represents the main pro-inflammatory driver and *Cdkn1a* directs apoptotic processes. Although genes in the coagulation network had mostly lower fold changes in comparison to the other genes in other sets, it contained some genes of interest such as *C3*, *Cfh*, *Timp1,* and *Timp3* that have all been connected to the development or progression of age-related macular degeneration (reviewed in^26^)^27^. The hubs of these genes may provide evidence for their connections to other regulated genes.

### Retinal adaptation to long-term normobaric hypoxia

**Figure 2:**
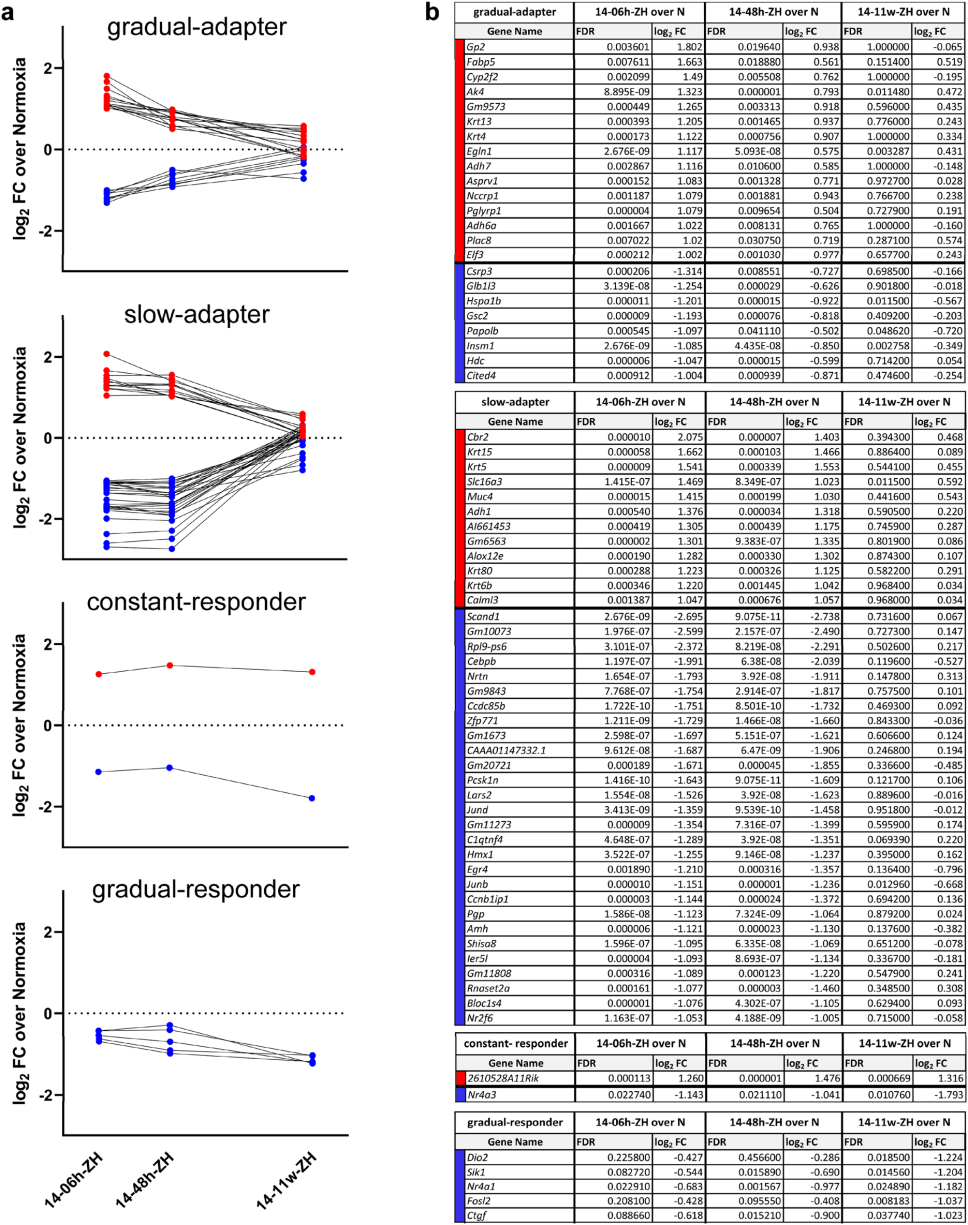
Adaptation of retinal gene expression profiles to normobaric hypoxia. (**a**) Gene expression pattern (log_2_FC over normoxia) after exposure to normobaric 14% O_2_ for 6 h, 48 h, or 11 weeks. Genes were filtered into 4 gene sets based on their expression at each time point. (**b**) Individual genes within each set. Up-regulated genes are shown in red and down-regulated genes in blue. FC: fold change; FDR: false discovery rate; N: normoxia.

Physiological systems change in response to chronically reduced oxygen levels to adapt to this environmental and cellular stress. Physiological adaptation begins immediately and is continuously adjusted during exposure and even beyond^28^. Since the mechanisms of molecular adaptation of the retina to low oxygen levels are fairly unknown, we compared gene expression at immediate (6 h) and acute (48 h) time points as well as after chronic (11 weeks) exposure to moderate (14% O_2_) normobaric hypoxia. We defined 4 groups of genes according to their expression pattern over time (Fig. 2a).

Genes of the first group, termed ‘gradual-adapter’, were significantly regulated immediately after exposure, but gradually returned to near-normoxic with a high FDR and a small log_2_FC at 11 weeks indicating that expression of these genes was no longer different from normoxic controls. Several members of this group of 23 genes (15 up- and 8 down-regulated genes) are known responders to variations in oxygen levels, including *Egln1, Adh7*, *Fabp5,* and *Cited4* (Fig. 2b).

The 'slow-adapter' group consisted of 40 genes characterized by their significant regulation at both the immediate (6 h) and the 48 h time points, but not at 11 weeks. A large proportion of the down- but not up-regulated genes in this group consisted of genes that encode proteins involved in gene regulation on the transcriptional (*Scand1, Cebpb, Ccdc85b, Zfp771, JunD, Hmx1, Egr4, Junb, Ier5l, Nr2f6*) or translational (*Rpl9-ps6*, *Lars1*) level (Fig. 2b). Within the up-regulated group, we identified several keratins (*Krt6b*, *Krt80*, *Krt15, Krt5*) and two genes involved in metabolic reactions (*Slc16a3* alias *Mct4* and *Alox12e*).

The group of 'constant responder' contained two genes, which were significantly regulated at all three time points. While the function of the up-regulated *2610528A11Rik* gene has not yet been determined, the down-regulated *Nr4a3* is involved in gene regulation.

The group of 'constant responder' contained two genes, which were significantly regulated at all three time points. While the function of the up-regulated *2610528A11Rik* gene has not yet been determined, the down-regulated *Nr4a3* is involved in gene regulation.

All genes of the fourth group ('gradual responder') showed a gradual down-regulation over time with the lowest expression levels after 11 weeks of hypoxic exposure. This group consisted of 5 genes, including the two transcription factors *Fosl2* and *Nr4a1* (nuclear receptor subfamily 4 group A member 1, or *Nur77*). *Nr4a1* is linked to circadian melatonin and dopamine release in the mouse retina^29^, diabetic retinopathy via NR4A1-dependent GFAT2 expression^30^, and regulation of apoptosis in hypoxic cancer tissues^31,32^. The third gene in this group*, Ctgf* (or *Ccn2*, cellular communication network factor 2), plays a role in cell adhesion and was shown to be involved in oxygen-induced retinopathy-related neovascularization^33^. *Dio2*, the main regulator of thyroid hormone signaling in the retina, has been linked to cone viability and was found to be expressed at elevated levels in degeneration models^34^. *Sik1* (Salt inducible kinase 1) was shown to be up-regulated in the genioglossus in the absence of *Hif1*^35^ and to play a crucial role in the *Sik1-Mef2-Bdnf* neurotrophic signaling pathway. Interestingly, *Nr4a1* is among the target genes of the *Sik1-Mef2-Bdnf* pathway^36^.

### Comparing the normobaric to the hypobaric hypoxic response

**Figure 3:**
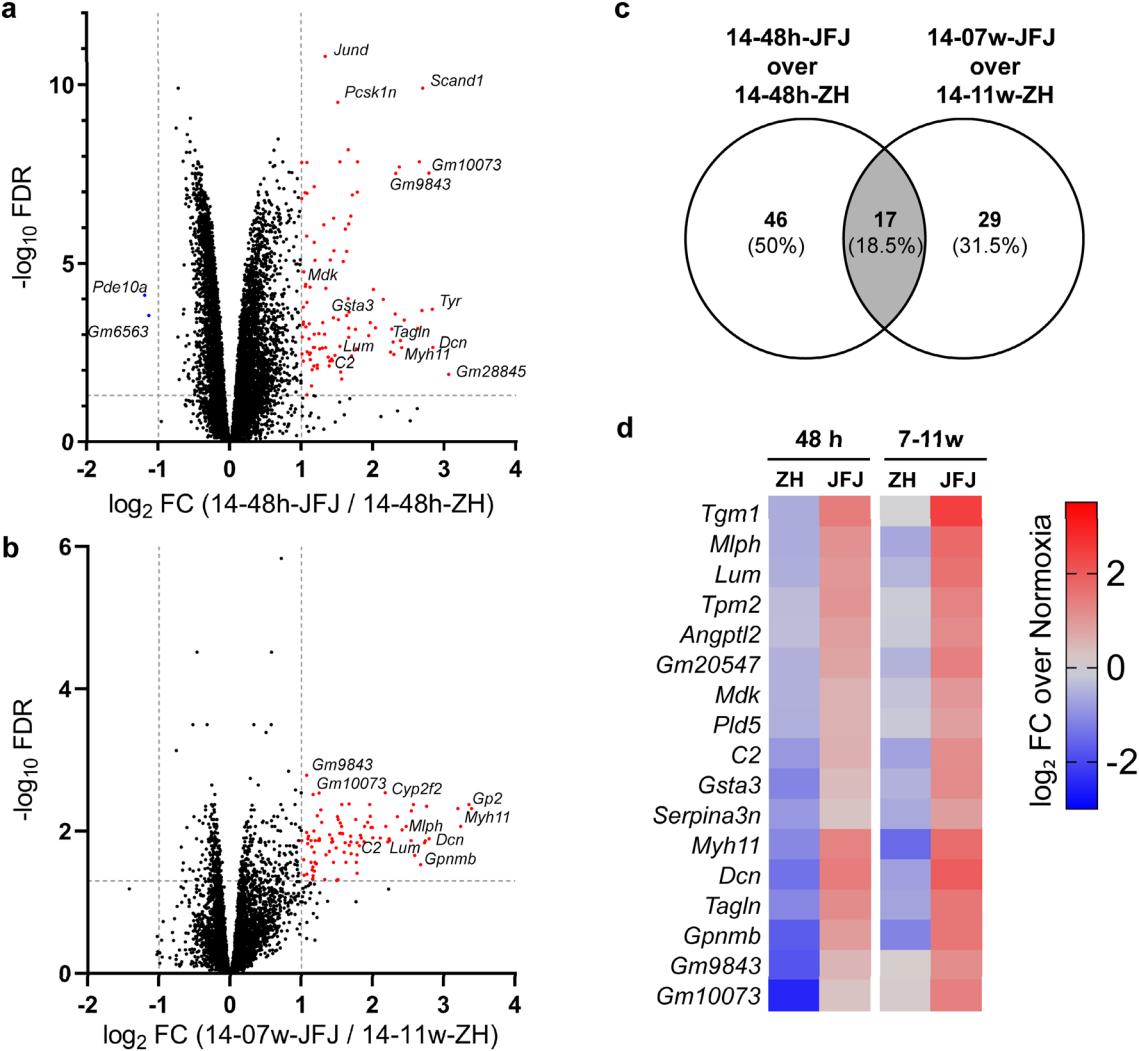
Differentially expressed genes after exposure to normobaric or hypobaric hypoxia. Volcano plots representing up-regulated (red) and down-regulated (blue) genes comparing (**a**) acute exposure (14-48 h-JFJ to 14-48 h-ZH) and (**b**) prolonged exposure (14-07w-JFJ to 14-11w-ZH) to hypobaric and normobaric hypoxia. Threshold limit lines are set at FDR of 0.05 and log_2_FC of ± 1. (**c**) Venn diagram showing differentially regulated genes at each time-point and their overlap (grey). (**d**) Heatmap of the 17 shared genes identified in (c). Shown are up- (red) and down- (blue) regulated genes as log_2_ fold change (FC) over normoxia in the short- or long-term groups as indicated.

In addition to a decrease in inspired oxygen (normobaric hypoxia), hypoxia occurs at high altitudes because of reduced barometric pressure (hypobaric hypoxia). Long term hypobaric hypoxia at high altitude activates physiological pathways similar, but not identical, to pathways activated by normobaric hypoxia (reviewed in^37^). However, potential differences in the regulation of gene expression in the retina in response to normobaric and hypobaric hypoxia have not been investigated. Therefore, we compared the transcriptomes of two groups of mice exposed to 14% normobaric hypoxia with two hypobaric groups exposed to reduced barometric pressure corresponding to approximately the same O_2_ levels (14%). The groups were exposed to either condition for a short (48 h) or a long time (7–11 weeks) (Table 1). The pairwise analysis identified 103 genes at 48 h and 98 genes at 7–11 weeks that were differentially regulated (Fig. 3a,b). 54 of these genes were common to both, the short-term and long-term comparison resulting in a total of 147 individual genes identified as being regulated in this analysis. Since hypobaric retinas had to be isolated without a microscope, samples were controlled for potential contamination by the RPE. For each of the 147 identified genes, fragments per kilobase of transcript per million mapped reads (FPKM) values (log_10_(FPKM + 1) were plotted for all conditions and compared to those of *Rpe65* as an RPE-specific marker gene (see Fig. S4 for examples). A correlation coefficient of ≥ 0.5 to the presence of *Rpe65* mRNA was taken as an indication that a significant portion of the mRNA detected in the samples was likely due to RPE contamination. These genes (38) were excluded from further analysis. The remaining 63 genes at 48 h and 46 genes at 7–11 weeks (Fig. 3c; Supplementary Dataset 4) did not correlate to *Rpe65* levels and were considered to be genuinely differentially regulated in the retina by normobaric and hypobaric hypoxia. Seventeen of these genes were regulated at both time points, suggesting that they may be of strong importance for the cellular response to reduced ambient air pressure (Fig. 3c,d). Surprisingly, all of these 17 genes were expressed at higher levels in hypobaric hypoxia (Fig. 3d). Five of the genes (*Lum*, *Angptl2*, *C2*, *Dcn*, *Gpnmb*) belong to a group of glycan-related genes that has been linked to angiogenesis^38^ and regulation by hypoxia^39^. Three genes (*Tpm2*, *Myh11*, *Tagln*) may be related to cellular filaments, and *Mdk* (midkine) and *C2* (complement C2) are connected to retinal degeneration and AMD, respectively.

### Hypoxic regulation in the retinal pigment epithelium

Studies with ascending probands have shown that retinal blood flow velocity increased at high altitude, ensuring a constant oxygen delivery to the inner retina. Choroidal perfusion, however, responded with a slight delay, and oxygen delivery capacity dropped within the first ascent to high altitude^17^. Since oxygen diffuses from the choroidal capillaries through the RPE to reach the outer retina, RPE cells may be good indicators of choroidal oxygen delivery. Thus, we investigated the expression of candidate genes in RPE/choroid (referred to as eyecup) in all groups of mice by semiquantitative PCR.

**Figure 4:**
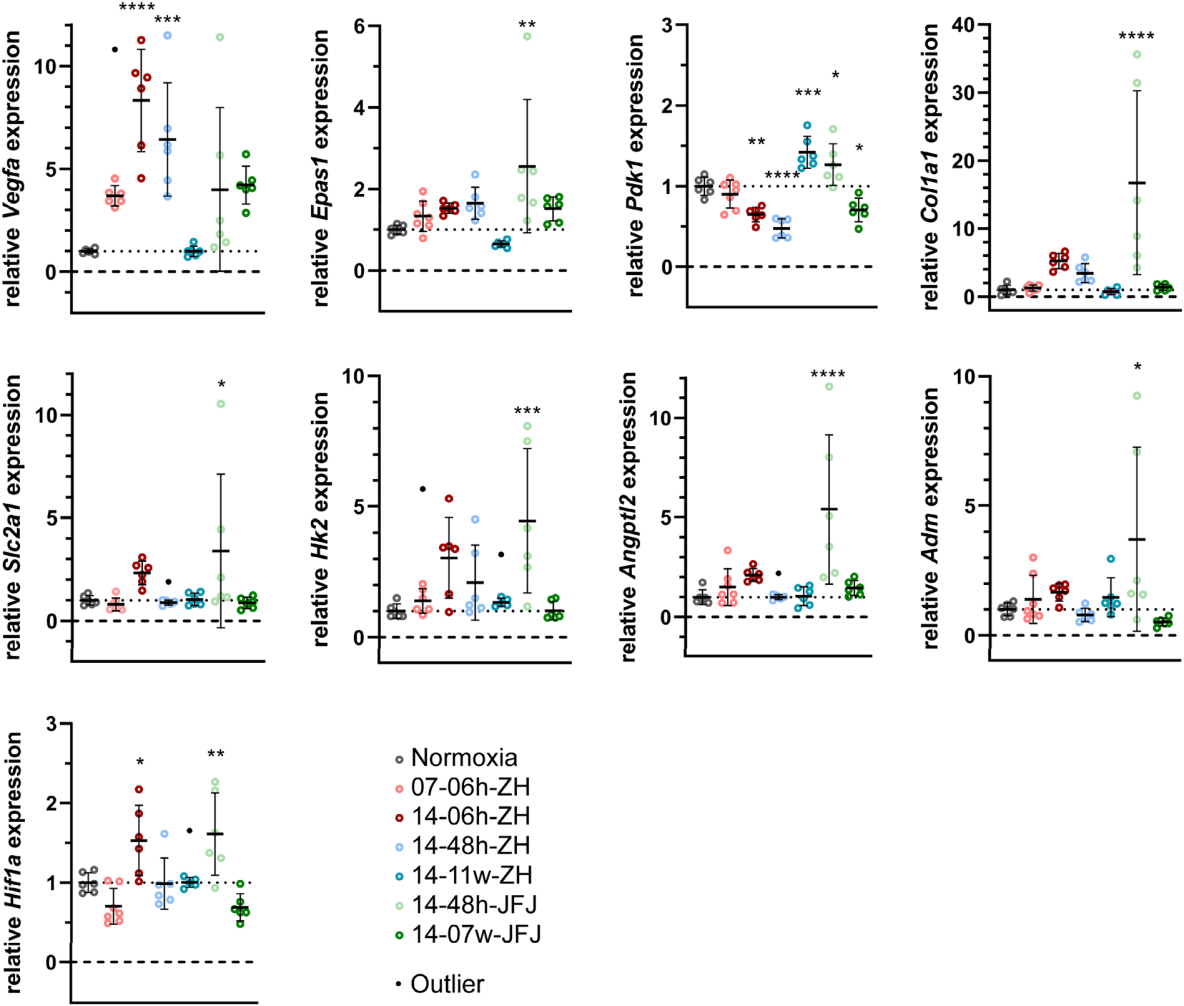
Differentially expressed genes in the RPE. Expression of genes related to the hypoxic response (*Hif1α, Epas1, Slc2a1,* and *Adm*), glycolysis (*Pdk1, Hk2*), vascular perfusion (*Vegfa, Angpt2*), and choroidal connective tissue (*Col1a1*) was tested in eyecups by semiquantitative real-time PCR for all hypoxic conditions, as indicated. Expression levels were normalized to *Actb* and are shown relative to normoxic controls, which were set to 1 (dotted line). Shown are means ± SD of n = 6. Outliers detected by Grubb’s test (α = 0.01) are indicated as black dots and were excluded from statistical analysis. Significant differences of each condition to control (normoxia) was tested by one-way ANOVA with Dunnett’s multiple comparison test *p < 0.05; **p < 0.01; ***p < 0.001; ****p < 0.0001.

We report an increase of *Vegfa* expression in the eyecups of all groups (3.7-fold in 07-06 h-ZH; 8.3-fold in 14-06 h-ZH; 6.4-fold in 14-48 h-ZH, fourfold in 14-48 h-JFJ and 4.2-fold in 14-07w-JFJ mice), except for mice exposed to chronic normobaric hypoxia (14-11w-ZH) (Fig. 4). Increased *Vegfa* gene expression was likely an attempt of the RPE to increase VEGFA secretion to improve oxygen delivery by enhancing choroidal circulation. Although *Epas1* was regulated to a much lower extent than *Vegfa*, the two genes showed a similar overall pattern (Fig. 4), indicating a possible role of HIF2A (encoded by *Epas1*) in regulating *Vegfa* expression in the RPE, as suggested by others^40^. In accordance with its role in choroidal neovascularization, *Vegfa* was up-regulated mainly in the eyecup but not so much in retinal tissue (Fig. 4, Table S2). Other genes connected to hypoxia were less strongly regulated. Except for *Pdk1,* none of the genes investigated was differentially expressed at the 7–11 w time points. *Col1a1* was included in the analysis because it was reported to be among the top regulated genes in the hypoxic RPE^40^. It is interesting to note that in contrast to the retina (see, e.g.^20^), gene expression in eyecups seemed more strongly regulated when the mice were exposed to mild (14%) than to severe (7%) hypoxia. Not only *Vegfa,* but also *Slc2a1, Hk2, Col1a1, Angpt2, Adm,* and *Hif1α* showed higher expression levels in the 14-06 h-ZH than in the 07-06 h-ZH group (Fig. 4), which may indicate that RPE and retina respond differently to a specific level of hypoxia.

### Photoreceptor segment shortening in hypoxic conditions

**Figure 5:**
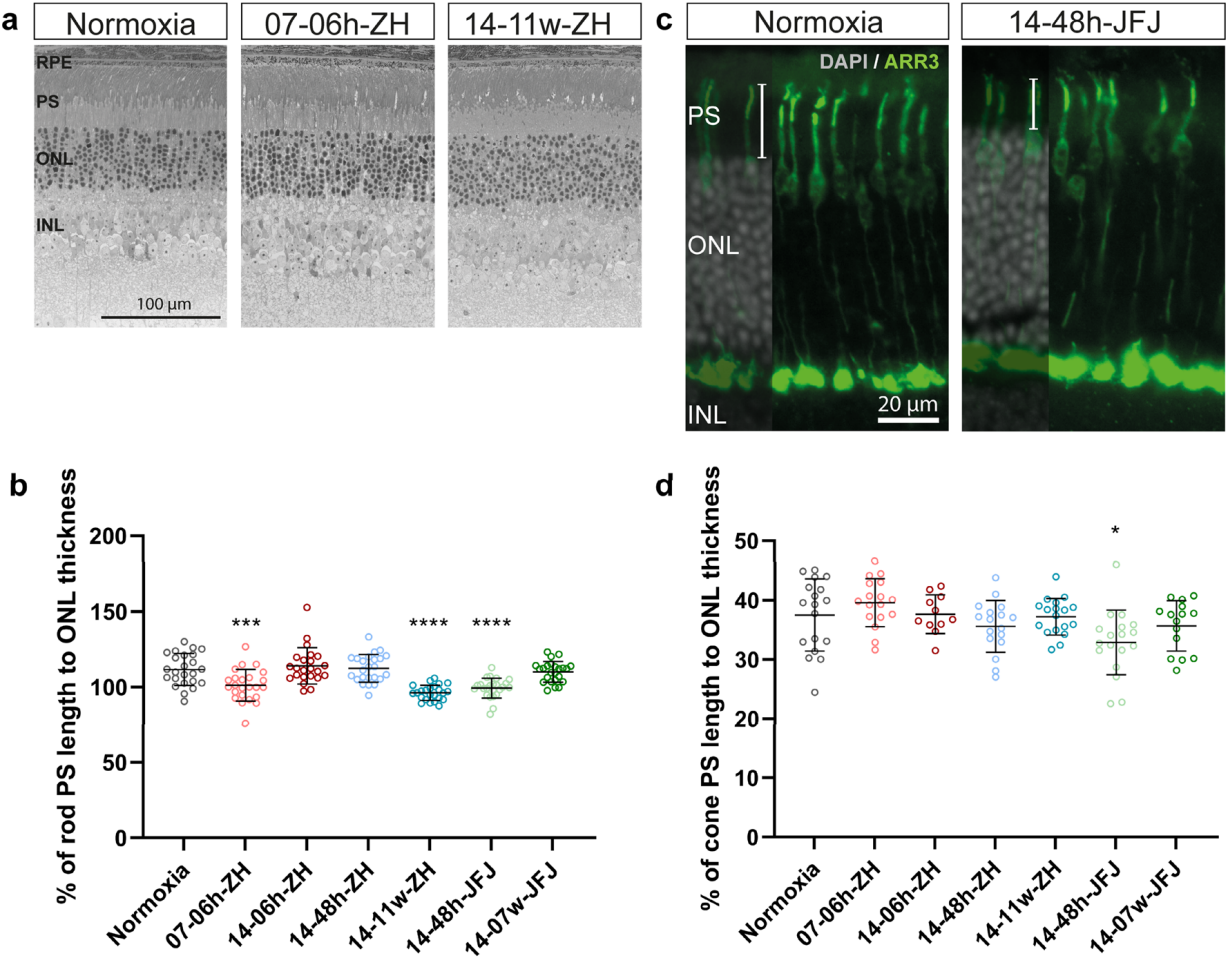
Photoreceptor segment lengths in hypoxia. (**a**) Representative retinal morphologies and (**b**) graph showing the ratio (%) of rod photoreceptor segment length to outer nuclear layer (ONL) thickness for indicated hypoxic conditions. (**c**) Representative immunostaining for cone arrestin (ARR3, green) and (**d**) the percentage of cone photoreceptor segment length to outer nuclear layer (ONL) thickness. Cell nuclei were counterstained with DAPI (grey). One-way ANOVA with Dunnett’s multiple comparison test was used to compare each condition to control (normoxia) *p < 0.05; ***p < 0.001; ****p < 0.0001. RPE: retinal pigment epithelium; PS: photoreceptor segments; ONL: outer nuclear layer; INL: inner nuclear layer; GCL: ganglion cell layer. Scale bars as indicated.

The retina in hibernating animals, like the ground squirrel, is exposed to reduced oxygen availability along with decreased metabolic activity during hibernation. The retina adapts to this situation by shortening and remodeling cone outer segments in the torpid state^41–43^. Based on these observations, we investigated outer nuclear layer (ONL) thickness and the length of rod and cone segments in the retinas of the different experimental groups. Rod segment length showed a significant shortening by approximately 10% in mice exposed to acute normobaric (07-06 h-ZH; 101.25 ± 10.44 μm; *p* = 0.0006), chronic normobaric (14-11w-ZH; 96.3 ± 4.97 μm; p < 0.001), and short hypobaric (14-48 h-JFJ; 99.39 ± 6.57 μm; *p* < 0.001) hypoxia in comparison to normoxia (111.61 ± 10.6 μm) (Fig. 5a,b). Cone segment lengths, however, were similar in all groups, except for a shortening in acute hypobaric mice (14-48 h-JFJ; 32.91 ± 5.46 μm; *p* = 0.0165) compared to normoxic mice (normoxia; 37.51 ± 6.09 μm) by about 12% (Fig. 5c,d). The ONL thickness was not significantly different between groups (Fig. S5).

## Discussion

Limited oxygen supply to the retina is a pathological situation that contributes to the development of blinding diseases including diabetic macular edema, wet AMD^44,45^, and potentially also dry AMD^5–8,46,47^. However, little is known about the response of the retina to acute or chronic hypoxia or to hypoxia at high altitude. Thus, we evaluated retinal expression of genes in response to different hypoxic conditions. Short time exposure to 7% O_2_ was chosen as this treatment was previously shown to be neuroprotective^20^. Furthermore, 14% O_2_ was selected because this concentration can be mimicked at high altitudes and thus by hypobaric conditions. The exposure periods of 2 days and 7–11 weeks, respectively, allowed comparison of acute and chronic responses and determination of the potential capacity of the retina to adapt to these conditions.

The retinas of mice exposed to the lowest oxygen levels (07-06 h-ZH) showed the highest number of differentially expressed genes among all six hypoxic groups. Regulation of many of the DE genes, including *Cdkn1a*, *Ttr*, *Bcl2l10*, *ApoldI*, *Mt2,* and others, was in accordance with an earlier microarray-based study^48^ validating our system. In our study, the most strongly induced genes were *H1foo* (oocyte-specific histone H1), *Mt2* (metallothionein 2), and *Apln* (apelin). All three genes have been connected to retinal pathophysiology associated with changes in oxygen levels. *H1foo* was found at increased expression levels in a mouse model of oxygen-induced retinopathy^49^, *Mt2* was attributed a role in choroidal neovascularization^50^, and *Apln* was implicated as a potential factor associated with hypoxia-induced retinal angiogenesis^51^. It is not surprising that the supreme enriched gene set was hypoxia (Fig. 1c, Supplementary Dataset 3). Another captivating, highly enriched network identified was glycolysis. Hexokinase is a key regulatory enzyme for glycolysis, which is highly expressed in photoreceptors^52^. The mRNA for isoform 2 (*Hk2*) was among the top regulated genes in this network, pointing towards an increased glycolysis. Such a glycolytic increase is further supported by the increased expression of *Ldha* (Fig. 1c, Supplementary Dataset 3), potentially leading to an elevated lactate production. Lactate might then be handled by MCT4, a lactate transporter across the plasma membrane encoded by the highly upregulated *Slc16a3* gene in this network. Our data set of genes involved in the acute response to strong hypoxia could be used as reference for studies of ischemic stroke, sleep apnea, and other conditions characterized by a sudden drop of oxygen levels, which can lead to visual disturbances (reviewed in^53,54^).

In contrast to acute and severe hypoxia, our data set on differentially expressed genes in long-term, moderate normobaric hypoxia aimed to identify adaptive gene expression patterns that may be relevant to physiological conditions in the aging eye, where reduced choroidal blood flow and age-related tissue alterations can cause mild but sustained hypoxia in the RPE and retina. To understand alterations and potential adaptive mechanisms under such conditions, we followed the expression levels of individual genes over time and categorized DE genes into 4 groups.

The group of gradual adapters represented genes which expression gradually returned to basal levels by 11 weeks of exposure (Fig. 2). Several of these genes (*Elf3, Csrp3, Gsc2, Insm1, and Cited4*) are involved in transcriptional regulation*.* Except for *Elf3*, all of these transcriptional regulators were initially downregulated, suggesting generally reduced transcriptional activity as an early retinal response. In contrast, genes involved in energy metabolism regulation, such as *Fabp5* (fatty acid-binding protein 5) and *Ak4* (adenylate kinase 4), were upregulated at the early but not late timepoint. *Fabp5* is involved in fatty acid uptake, transport, and metabolism. The upregulation of *Fabp5* in animal models with acute oxygen deficiency such as neurotrauma and cerebral ischemia^55,56^, as well as in cancer^57^, suggests that it may compensate for energy stress and prevent apoptosis. *Ak4* regulates cellular ATP levels and is thus directly involved in controlling energy metabolism. Elevated *Ak4* levels during early hypoxia may contribute to protection against cell death^58^. *Hspa1b* (heat shock 70 kDa protein 1B) is another interesting gene that was initially downregulated, but its expression later returned to basal levels. *Hspa1b* encodes a heat shock protein that protects other proteins from stress-induced degradation^59,60^. The downregulation of *Hspa1b* may support cellular systems that control global protein load^61^, potentially optimizing cellular energy demand. The relevance of *Hspa1b* in hypoxic ocular tissues is further exemplified by a polymorphism in *HSPA1B,* which is associated with the development of glaucoma^62^. Another downregulated gene associated with retinal disease is *Glb1l3* (Galactosidase beta 1 like 3), which has been implicated in age-related metabolic stress and found to be reduced in *Rpe65* knockout mice, connecting it to the pathophysiology of Leber congenital amaurosis^63^. Together, our data suggest that the group of gradual responders includes genes that help ignite a quick response to cope with the adverse conditions by regulating genes that restore energy metabolism or reduce energy-consuming processes such as gene and protein expression.

In the group of slow adapters, we identified several downregulated genes that are involved in the development and/or progression of retinal degeneration (*Cebpb, Egr4,* and *Ier5l*) or apoptosis (*Nrtn, Ccdc85b, Junb* and *Jund*). In contrast, four keratin genes (*Krt15, Krt5, Krt80,* and *Krt6b*) that support structural cell and tissue integrity were upregulated. This may indicate that after the acute reaction to hypoxia that aims to maintain energy homeostasis (see above), retinal cells may attempt to prevent cell death and support or maintain structural tissue integrity in a more prolonged, slowly adapting response that returns to basal levels only later.

Genes in the groups of constant and gradual responders were mostly downregulated. While decreased *Dio2* expression has been shown to improve cone survival, likely through modulation of cellular thyroid hormone signaling^34^, decreased *Nr4a1* and *Nr4a3* levels may modulate the HIF1 pathway. Since NR4A1 can stabilize HIF1A at the protein level by increasing its transcriptional activity^64^ and NR4A3 has been proposed as a downstream effector of HIF1 signaling^65^, reduced expression of the two nuclear receptors may down modulate HIF1 activity and thus contribute to the adaptation process. As the gradual responder genes do not return to basal levels even after prolonged exposure to hypoxia, they might represent key regulators of the cellular adaptation to long-term hypoxia.

The general cellular response to hypoxia through transcriptional regulation by HIF1 and HIF2 is well described (reviewed in^66^). Since these transcription factors are mostly regulated on the protein level, their transcripts were not identified in our transcriptomic approach. However, we found several other genes across all groups that are directly or indirectly linked to gene regulation. Since most of them (*Cebpb*, *Cited4*, *Nr4a1*, *Nr4a3*, *Insm1*, *Scand1*, *Rpl9*, *Zfp771*, *Egr4*, *Junb*, *Jund*, *Fosl2* and *Papolb*) were down- and only a few (*Egln1*, *Elf3*) were upregulated, our data may indicate that retinal cells might compensate for increased HIF-mediated gene expression by downregulating other regulatory genes or proteins during adaptation to hypoxia, likely in an attempt to save energy and optimize metabolism under these conditions.

In addition to analyzing retinal mRNA levels during short and long-term hypoxia, we compared retinal gene expression of mice exposed to normobaric and hypobaric hypoxia at high altitude. This data set might be of particular interest for studies on high-altitude retinopathy and retinal hemorrhage^13,19^. Among the 17 genes that were expressed at higher levels at both time-points during hypobaric hypoxia (Fig. 3c,d), we identified *Lum* (Lumican) and *Dcn* (Decorin), members of the small leucine-rich proteoglycan family. Both contribute to the formation of the extracellular matrix (ECM) in sclera and cornea^67^, but have also been detected in the retina^68,69^. While LUM is associated with the development of myopia, DCN has multiple functions including regulation of inflammation, angiogenesis^70^, and as neurotrophic factor during retinal differentiation^71^. We hypothesize that increased expression of these genes may lead to modifications of the ECM composition, potentially affected by changes in intraocular pressure at high altitude^72,73^. In support of this hypothesis, three other genes (*Tpm2, Myh11,* and *Tagln*) that are connected to filaments and thus to intracellular structural integrity were also upregulated in hypobaric hypoxia. Tropomyosin 2 (*Tpm2*), for example, stabilizes actin filaments and regulates structural and dynamic properties (reviewed in^74^). Such potential changes in the ECM composition and structural integrity of filaments could create conditions that facilitate the shortening of cone and rod photoreceptor segments, as observed under three conditions, including short-term hypobaric hypoxia (Fig. 5b,d).

Shortening of photoreceptor outer segments was also observed in ground squirrels during hibernation^41–43,75^, which can be described as a “natural” form of chronic hypoxia. Analysis of the expression levels of genes involved in photoreceptor inner/outer segment formation revealed two up-regulated genes in the 07-06 h-ZH group, *Kif17* and *Mertk*, which are connected to photoreceptor turnover and homeostasis^76,77^. This raises the possibility that the shortening of segments in hypoxia may be associated with an altered balance between disc biogenesis and shedding. Although further experimentation is needed to test the potential effect of hypoxia on these processes, it seems clear that even prolonged exposure to moderately reduced oxygen levels does not lead to drastic changes in the macroscopic architecture of the retina or individual photoreceptors in mice.

## Conclusion

This study provides a comprehensive overview of the transcriptomic response of the retina to normobaric and hypobaric hypoxia. An overall view of the adaptation of retinal cells to sustained hypoxia is provided, and the differences in the consequences for gene expression after exposure to high altitude or normobaric hypoxia are highlighted. Gene clusters of the acute response to hypoxia may serve as a reference for situations in which acute changes in oxygen levels contribute to pathology (e.g. stroke), whereas genes identified in the chronic, moderate hypoxia groups may support research of diseases characterized by reduced oxygenation of the retina over a prolonged period, such as the slowly developing dry AMD.

## Material and methods

### Mice

This study is reported in accordance with the ARRIVE guidelines. Animal maintenance and experimentation adhered to the regulations of the Cantonal Veterinary Offices of Kanton Bern and Zurich, Switzerland and the ARVO Statement for the use of Animals in Ophthalmic and Vision Research. The protocol was approved by the veterinary authorities of Kanton Zurich and Bern (license numbers: ZH080/19, ZH214/17). Mice assigned to the low-altitude groups were housed in the animal facility of the University of Zurich at 408 masl with a 14/10 h light/dark cycle. A total of 52 C57BL/6J mice were included in the study providing material for sequencing, immunofluorescence, and morphology evaluation. Animals assigned to the high-altitude groups were transported to the high-altitude research station on the Jungfraujoch ((JFJ) 3450 masl) in a single journey of 270 min duration and maintained with a 12/12 h light/dark cycle. All animals had access to food and water ad libitum and cages included environmental enrichment. Average light intensities were between 60 to 150 lx, depending on the position of the cages in the facilities. The normoxic control group included 6 males and 4 females, and the experimental groups 8 males and 8 females for high altitude (hypobaric hypoxia) and 15 males and 11 females for normobaric hypoxic conditions. 42 mice (n = 6, for each group) were used for RNA sequencing. No gender specific comparison was performed during this study.

### Exposure to hypoxia

Mice (n = 6) were assigned to control groups (normoxia) or 6 different hypoxic conditions, defined by the following terminology ‘oxygen saturation – time spent in hypoxia (duration)—location of experiment’ (Table 1). For all experiments performed in Zurich (ZH), mice were placed in their home cage in isobaric hypoxia chambers and exposed to hypoxia for 6 h, 48 h, or 11 weeks. Briefly, oxygen concentration was decreased gradually by 2% every 10 min by altering the oxygen:nitrogen ratio until either 14% or 7% O_2_ was reached. For hypobaric experiments performed at high altitude (JFJ), mice were exposed to the environmental air pressure of approximately 64.65 kPa (slightly fluctuating depending on weather and time of the year), which decreases the ambient partial pressure of oxygen from 21.2 kPa (sea level) to 13.7 kPa (reviewed in^78^).

### Hematocrit and hemoglobin measurements

Mice were euthanized with an overdose of pentobarbital, and blood samples were taken by cardiac puncture with a 30G needle attached to a 1 mL heparinized syringe. Subsequently, the blood was transferred to heparinized microcapillaries (Micro Hematocrit Capillaries, Hecht Assistant, Germany) and centrifuged for 5 min at 1 × 10^4^ rpm (Hematocrit 20 centrifuge, Hettich, Germany). Hemoglobin measurements were performed using ABL800 (Radiometer RSCH GmbH, Switzerland) according to manufacturer’s instructions, or measurements were performed using a portable device (LP 420 Laborphotometer, Dr.Lang, Germany) for samples collected at JFJ.

### RNA Isolation, RNA sequencing, and real-time PCR

Retinas were isolated through a slit in the cornea and snap-frozen in liquid nitrogen. For isolation of RNA from the RPE, the eyecup (EC) containing RPE was dissected, cleaned from excessive connective tissue, and snap-frozen. If transportation was necessary, retinas were kept on dry ice before they were stored at − 80 °C. RNA was isolated using an RNA isolation kit (Nucleo Spin RNA, Macherey–Nagel GmbH & Co.KG, Düren, Germany) with an additional, on-column DNase I treatment according to the manufacturer’s instructions. Samples with high-quality RNA (RQN ≥ 6.9) measured with ProSize Data Analysis Software (Version 4.0, www.agilent.com/en/product/automated-electrophoresis/fragment-analyzer-systems/fragment-analyzer-systems-software/fragment-analyzer-software-1149185) and the Fragment Analyze Automated CE System (Advanced Analytical Technologies, Inc., Ankeny, USA) were used. One retina of each animal was used for sequencing data acquisition, whereas the contralateral eye was used either for immunofluorescence or morphologic evaluation. N = 6 per experimental group. The RNA samples were individually processed, and sequencing was performed on an Illumina NovaSeq (Illumina, Eindhoven, Netherlands) using a paired-end high-output sequencing kit (Illumina) at the Functional Genomics Center Zurich (FGCZ, University of Zurich, Zurich, Switzerland). GRC38p5 was used as the reference genome for all analyses (https://www.ncbi.nlm.nih.gov/assembly/GCF_000001635.25/). Supplementary Dataset 5 shows the acquired data for all 25,700 sequences in the reference genome set for all conditions.

For real-time PCR, first-strand cDNA synthesis was performed by M-MLV reverse transcriptase (Promega, Dübendorf, Switzerland) using 1 μg of RNA. Gene expression analysis was performed via semi-quantitative real-time PCR (QuantStudio 3, ThermoFisher Scientific, Bremen, Germany) with 10 ng of cDNA as template and PowerUp SYBR Green Master Mix (ThermoFisher Scientific). Primer pairs (Table S4) were designed to span large intronic regions and avoid known SNPs in mouse sequences. Gene expression was normalized to β-Actin (*Actb*), and relative expression was calculated by the comparative threshold cycle method (2^-ΔΔCT^)^79^. Grubb’s outlier test with α = 0.01 was performed to identify outliers.

### Bioinformatic analysis

Bioconductor package edgeR^80^ was applied for pairwise comparisons between the various conditions to normoxia. FastQC was performed for quality control. A gene set most likely representing contaminating genes from RPE or vitreous, and genes with strongly varying expression values across samples were excluded from downstream analysis (Table S3). Differentially expressed (DE) genes were defined by applying the Benjamini–Hochberg false discovery rate (FDR) method^81,82^ and setting the significance threshold to ≤ 0.05 and a log_2_ fold-change ≥ ± 1. FDR was chosen over Bonferroni correction for its greater power to detect true positives while still controlling for false positives at an acceptable level. Additionally, an FPKM filter was applied, and genes with more than 3 replicates in one group displaying an FPKM of 0 were excluded from the analysis.

Clustering and hierarchical heatmap visualization was performed in Rstudio (Package: ComplexHeatmap Version 2.8.0; R Version 3.6.0, www.R-project.org). Gene set enrichment analysis (GSEA) was performed using GSEA (Version 4.1.0, www.gsea-msigdb.org) software provided by UC San Diego, Broad Institute of Massachusetts Institute of Technology and Harvard University^83,84^. The analysis was conducted on pre-ranked gene list based on log_2_ fold-changes. The hallmark gene sets from Molecular Signatures Database (MSigDB, Version 7.2, www.gsea-msigdb.org^85,86^) were used to compare 07-06 h-ZH versus normoxic conditions.

The Cytoscape (Version 3.8.2, www.cytoscape.org) plug-in GeneMANIA (Version 3.5.2, www.genemania.org^87^) was used to generate gene co-expression networks based on expression data sets from publicly available databases.

### Morphology and immunofluorescence

For morphologic examination, mice were euthanized with either CO_2_ inhalation followed by decapitation or an overdose of pentobarbital. Eyes were marked at the nasal limbus, enucleated, and fixed in 2.5% glutaraldehyde in cacodylate buffer for 12–24 h at 4 °C. After trimming and dehydration in increasing concentrations of EtOH, eyes were postfixed in 1% osmium tetroxide and embedded in Epon 812 as previously described^88^. Dorso-ventral cross-sections (0.5 μm) cut through the optic nerve head were counterstained with toluidine blue and analyzed by light microscopy (Axioplan, Zeiss, Jena, Germany). Length of photoreceptor segments and thickness of the ONL were measured every 200 μm between 200–1200 μm ventral and dorsal of the optic nerve head using Adobe Photoshop CS6 ruler tool (Adobe Systems, Inc., San Jose, CA, USA) on reconstructed retinal panorama images^89^. Three measurements of ONL and photoreceptor segments were averaged per area. To account for potential variations in the fixation-induced shrinkage factor between eyes, photoreceptor segment lengths were expressed relative to the ONL thickness (Fig. S5) at each measured point along the retina.

For immunofluorescence, eyes were marked nasally and fixed in 4% paraformaldehyde (PFA) in phosphate buffer as described before^89^. After removal of the lens, eyecups were further fixed in 4% PFA for 2–24 h. After fixation, eyecups were cryoprotected in 30% sucrose (Sigma-Aldrich, St. Louis, MO, USA) overnight, embedded and frozen in freezing medium (O.C.T., Leica Biosystems Nussloch GmbH, Nussloch, Germany), and stored at − 80 °C until sectioning. Twelve micron thick dorso-ventral sections were blocked in phosphate buffer containing 3% normal goat serum (Sigma-Aldrich) and 0.3% Triton X-100 (Sigma) followed by incubation with Isolectin (GS-IB4-Alexa594, 1:250, I21413, Invitrogen) or the following primary antibodies in blocking solution overnight: rabbit anti-ARR3 (cone arrestin 1:1000, AB15282, Merck); mouse anti-GFAP (glial fibrillary acidic protein, 1:250, G3893, Sigma) and rabbit anti-AIF1 (allograft inflammatory factor 1 (also called IBA1), 1:500, 019-19741, Wako). After washing, sections were incubated for 1 h with corresponding secondary antibodies in blocking solution and counterstained with 4′,6-diamidino-2-phenylindole (DAPI). After mounting, slides were examined using a fluorescence microscope (Axioplan, Zeiss) and analyzed using the Adobe Photoshop CS6 ruler tool (Adobe Systems). Cone segments were measured every 200 μm from 400 to 1200 μm ventral and dorsal to the optic nerve head. The average values of 10 cones are given for each measurement. ONL thickness was determined by averaging 3 measurements every 200 µm as described above.

### Statistical analysis

Statistical analysis was performed using GraphPad Prism version 8.4.3 for Windows (GraphPad Software, San Diego, Ca, USA, www.graphpad.com). All data are presented as mean values ± standard deviation (SD). For qPCR analysis, significance was determined by one-way ANOVA (α = 0.05) against normoxic control with Dunnett’s multiple comparison post-test and additional Grubbˈs test (α = 0.01) to identify outliers. Significance test for hemoglobin and hematocrit analysis was performed with one-way ANOVA with Holm-Sidak’s multiple comparison test (α = 0.05).

## Supplementary Information

Supplementary Data 1.

Supplementary Data 2.

Supplementary Data 3.

Supplementary Data 4.

Supplementary Data 5.

Supplementary Information.

## Data Availability

All datasets generated during the current study are included in this manuscript and its Supplementary Datasets 1–5. The data discussed in this publication have been deposited in NCBI's Gene Expression Omnibus^90^ and are accessible through GEO Series accession number GSE173233 (https://www.ncbi.nlm.nih.gov/geo/query/acc.cgi?acc=GSE173233).
