# Supplementary material for: Transcriptomic analysis of the mouse retina after acute and chronic normobaric and hypobaric hypoxia
Source: Sci Rep. 2021 Aug 17;11:16666. doi: 10.1038/s41598-021-96150-9 (PMC8371159; doi:10.1038/s41598-021-96150-9)
Supplement: Supplementary file 6 — Supplementary Information. [file 41598_2021_96150_MOESM6_ESM.pdf]

Supplemental Material

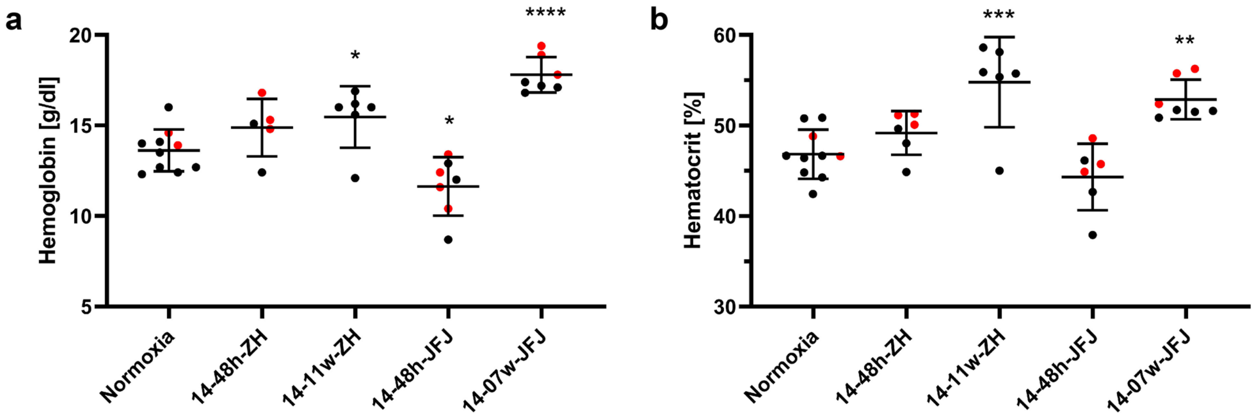

Figure S1| Hemoglobin and hematocrit levels in mice exposed to different hypoxic conditions.

a) Hemoglobin values in [g/dL]. **b)** Hematocrit in [%]. Shown are means ± SD, n≥5. Female samples are indicated in red. Significance tested with one-way ANOVA with Holm-Sidak's multiple comparison to normoxic group. *p<0.05; **p<0.005; ***p<0.0005 ****p<0.0001. Groups represent exposure to 14% O_2 for short (48 h) and long term (07-11 weeks) in normobaric (ZH) and hypobaric (JFJ) hypoxia.

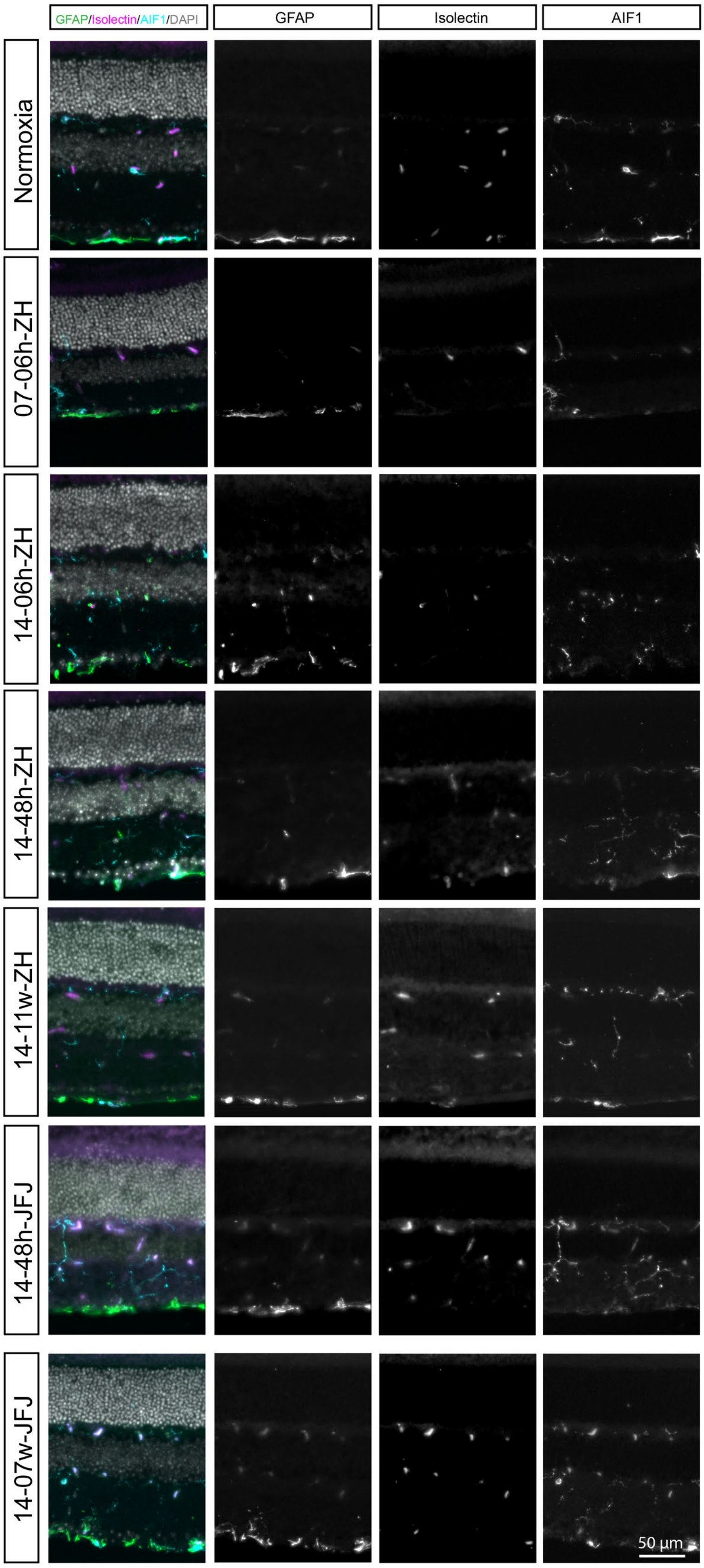

Figure S2| Immunofluorescence of markers for gliosis, blood vessels and, microglia/macrophages.

Immunostaining of glial fibrillary acidic protein (GFAP in green), isolectin as vascular stain (magenta), and allograft inflammatory factor 1 (AIF1 alias IBA1, blue) for microglia in indicated experimental groups. Cell nuclei were counterstained with DAPI (grey). Scale bar as indicated.

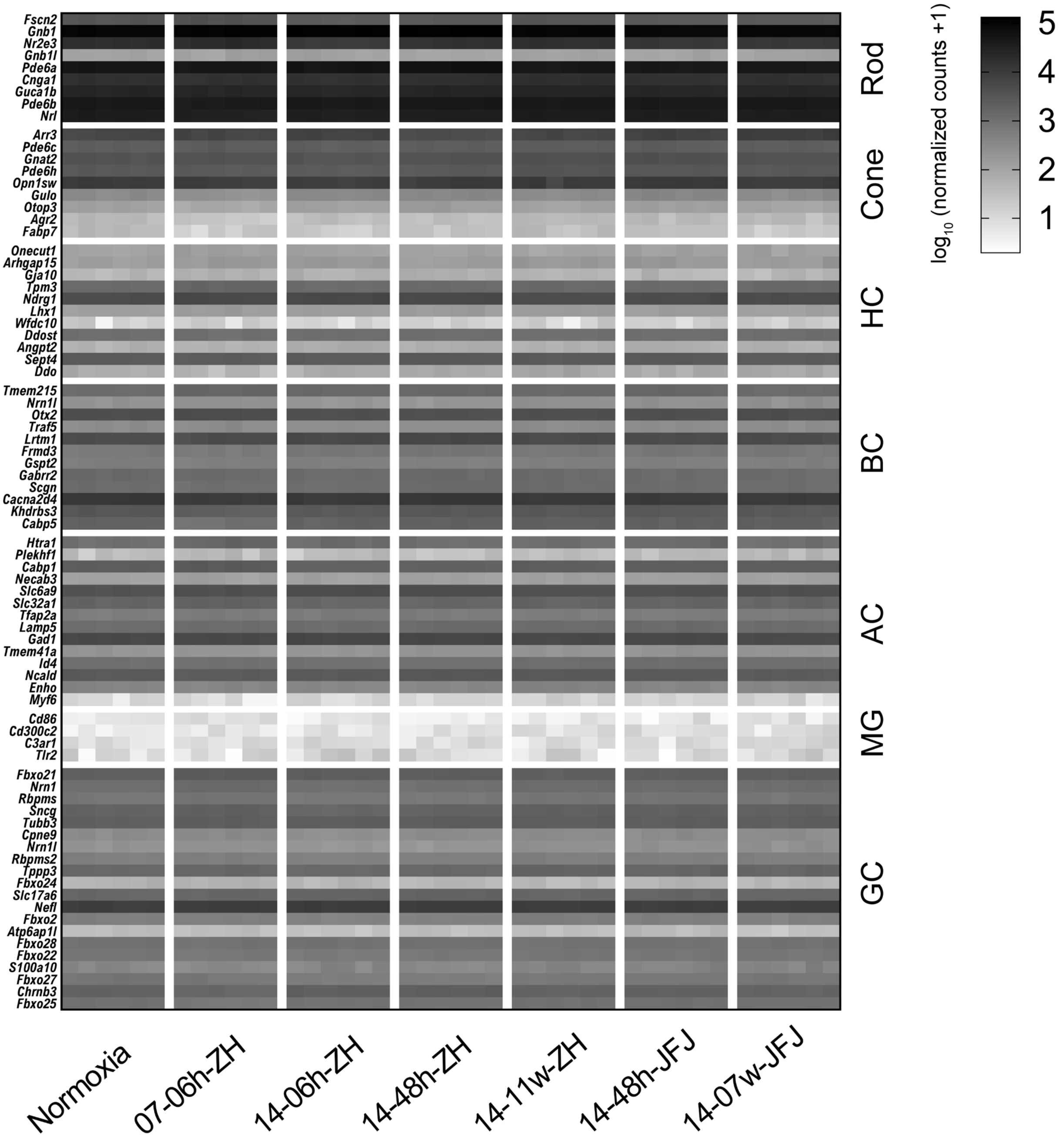

Figure S3| Heatmap of cell type-specific genes.

Plotted are $\log_{10}$(normalized counts+1). HC: horizontal cells; BC: bipolar cells; AC: amacrine cells; MG: microglia; GC: ganglion cells.

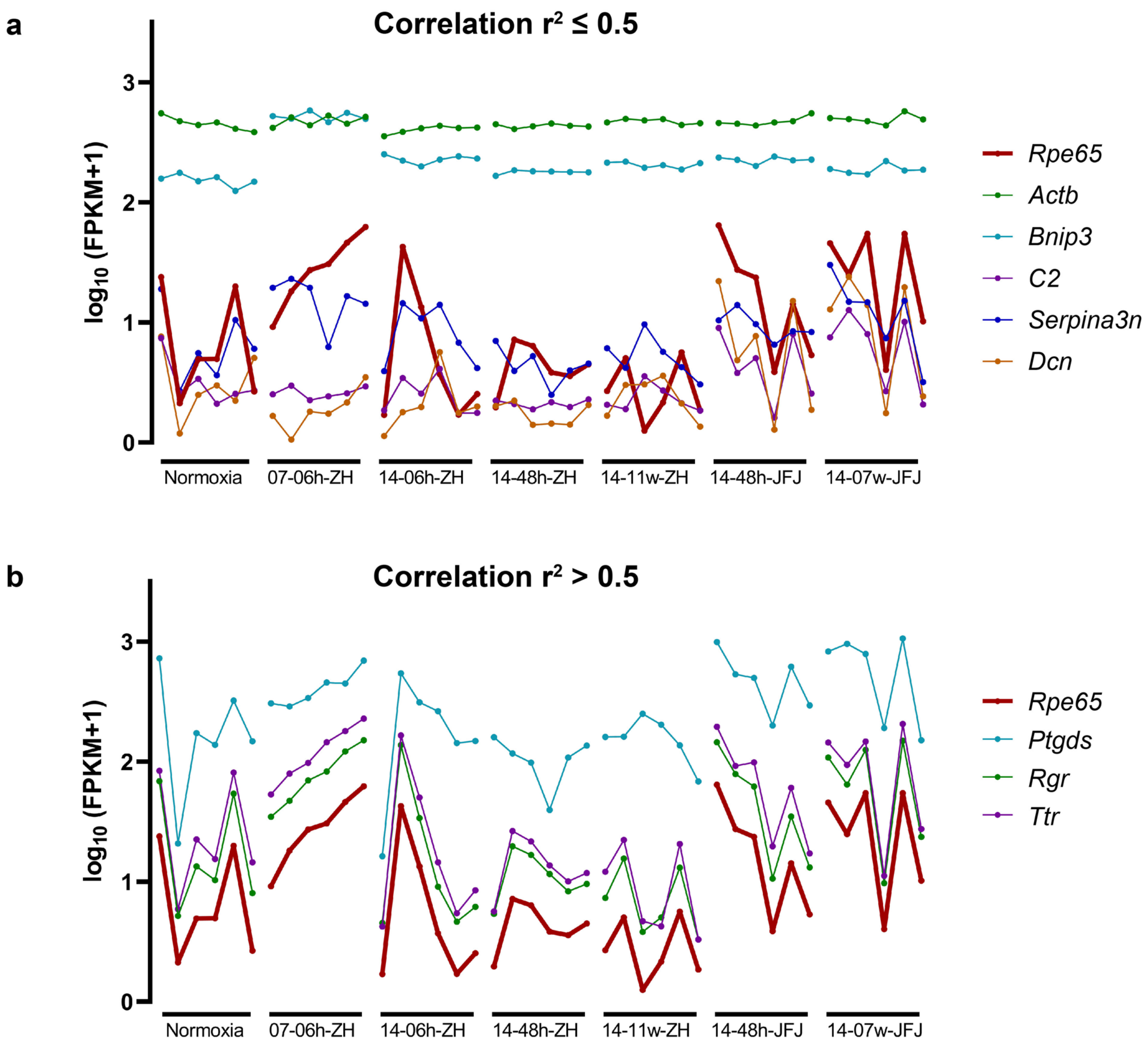

Figure S4| Correlation to *Rpe65* expression as an indicator of contamination with RPE transcripts.

a) Example genes which displayed a correlation coefficient $r^2 \leq 0.5$ to *Rpe65*. These genes are unlikely to result from an RPE contamination. Actin-beta (*Actb*) is displayed as a housekeeping gene, BCL2 Interacting Protein 3 (*Bnip3*) is exemplarily shown as a hypoxia regulated gene. **b)** Example genes which displayed a correlation coefficient higher than 0.5 to *Rpe65* expression, indicating that some of the signals were attributable to RPE contamination. The genes that had an $r^2 \geq 0.5$ were excluded from further analysis.

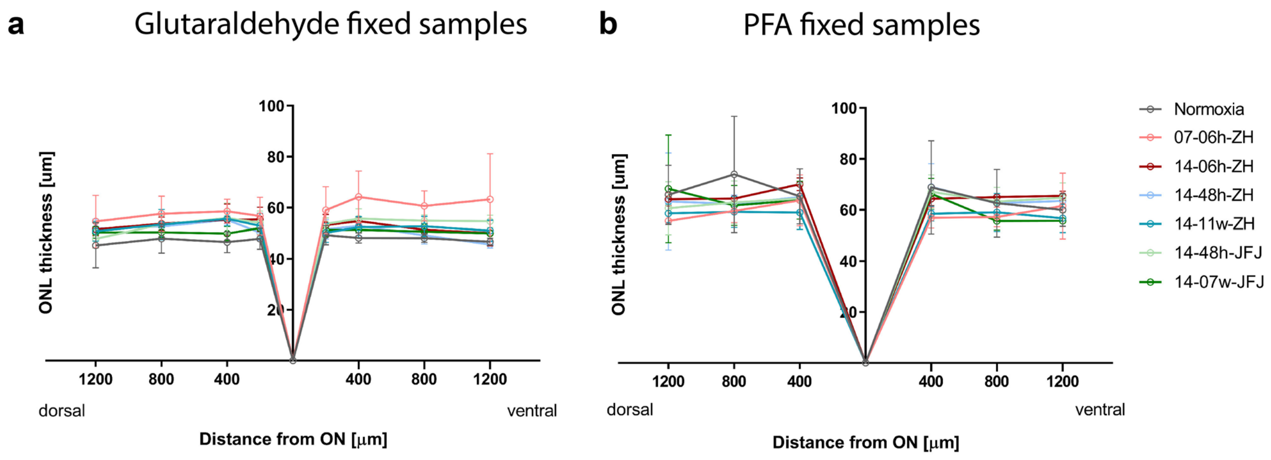

Figure S5 |Thickness of the outer nuclear layer under hypoxic conditions.

Spiderograms of outer nuclear layer (ONL) thickness measured at indicated points dorsal and ventral to the optic nerve head (ON) in different hypoxic conditions after **a)** glutaraldehyde fixation of samples for morphological evaluation and **b)** 4% PFA fixation of eyes serving for immunofluorescence analysis. ONL: outer nuclear layer; ON: optic nerve head; PFA: paraformaldehyde. Shown are means ± SD of n=6 per group.

Table S1 | Hemoglobin and Hematocrit values

Condition	Hemoglobin [g/dL] mean ± SD	Hematocrit [%] mean ± SD
Normoxia	13.62 ± 1.15	46.83 ± 2.71
14-48h-ZH	14.88 ± 1.58	49.17 ± 2.41
14-48h-JFJ	11.63 ± 1.61 *	44.32 ± 3.67***
14-11w-ZH	15.47 ± 1.7 *	54.79 ± 4.98
14-07w-JFJ	17.8 ± 0.98 ****	52.87 ± 2.19 **

Hypoxic groups were compared to normoxic controls. *: $p < 0.05$; **: $p < 0.01$; ***: $p < 0.001$; ****: $p < 0.0001$.

Table S2 | Correlation between RNA-Seq and qPCR gene expression in the retina

Gene name	FC RNA-Seq over Normoxia					
	07-06h-ZH	14-06h-ZH	14-48h-ZH	14-11w-ZH	14-48h-JFJ	14-07w-JFJ
Adm	5.56	1.65	1.35	1.23	1.55	1.30
Bnip3	3.50	1.57	1.22	1.33	1.45	1.21
Egln1	6.16	2.17	1.49	1.35	1.62	1.50
Slc2a1	2.01	1.52	1.23	1.09	1.42	1.33
Pdk1	2.91	1.85	1.32	1.20	1.48	1.30
Vegfa	1.83	1.00	0.95	1.09	1.21	1.06

Gene name	FC qPCR over Normoxia					
	07-06h-ZH	14-06h-ZH	14-48h-ZH	14-11w-ZH	14-48h-JFJ	14-07w-JFJ
Adm	23.22	3.26	2.38	1.29	5.27	3.59
Bnip3	2.20	1.00	0.62	1.20	0.81	0.82
Egln1	3.60	1.33	0.76	1.18	0.89	0.87
Slc2a1	0.95	0.84	0.55	0.96	0.78	0.82
Pdk1	1.49	0.80	0.48	0.95	0.83	0.71
Vegfa	1.13	0.76	0.63	0.94	0.83	0.69

Shown are fold changes (FC) over normoxic controls for RNAseq and qPCR data for each experimental condition. N=6.

Table S3 | Excluded gene list

Group	Genes excluded from analysis
Crystallins	*Cryab; Cryba1; Cryba2; Cryba4; Crybb1; Crybb2; Crybb3; Crybg1; Crybg2; Crybg3; Cryga; Crygb; Crygc; Crygd; Cryge; Crygf; Crygn; Crygs; Cryl1; Crym; Cryz; Cryaa*
Hemoglobins	*Hba-a1; Hba-a2; Hba-x; Hbb-bh1; Hbb-bh2; Hbb-bs; Hbb-bt; Hbb-y*
RPE-specific	*Dct; Lgsn; Lrat; Mlana; Pmel; Rpe65; Slc45a2; Tyrp1*

Table S4 | Primers used for qPCR

Gene	forward Primer (5'- 3')	reverse Primer (5'- 3')
Hif1a	TCATCAGTTGCCACTTCCCCA	CCGTCATCTGTTAGCACCATC
Epas1	GGAGCTCAAAAGGTGTCAGG	CAGGTAAGGCTCGAACGATG
Adm	TCCTGGTTTCTCGGCTTCTC	ATTCTGTGGCGATGCTCTGA
Slc2a1	CAGTGTATCCTGTTGCCCTTCTG	GCCGACCCTCTTCTTTCATCTC
Hk2	CCTGGTTTCAAAGCGGTCGG	TACTGGTCAACCTTCTGCACTTG
Col1a1	TGTTCAGCTTTGTGGACCTC	TCAAGCATACCTCGGGTTTC
Pdk1	GTTGAAACGTCCCGTGCT	AGTCTCTCGACGGATTCTGT
Angptl2	TCGCTGGTGAAGAGTCCAAC	GACCACATGCGTCAAACCAC
Vegfa	ACTTGTGTTGGGAGGAGGATGTC	AATGGGTTTGTCGTGTTTCTGG
Bnip3	CCTGTCGCAGTTGGGTTC	GAAGTGCAGTTCTACCCAGGAG
Egln1	GCAGCATGGACGACCTGAT	CAACGTGACGGACATAGCCT

Supplementary Dataset 1 (Supplementary Dataset 1.xlsx)

Differentially expressed genes (FDR ≤ 0.05) in each group over normoxia. A detailed list of genes shown in Fig. 1a. Given are the official gene name, ensembl number (Identifier), gene description, the log_2FC over normoxic controls, FDR and p-Value. Individual tabs show values of each DE gene over normoxia for each hypoxic condition: Tab1: 07-06h-ZH; Tab2: 14-06h-ZH; Tab3: 14-48h-ZH; Tab4; 14-11w-ZH; Tab5: 14-48h-JFJ; Tab6: 14-07w-JFJ. FC: fold change; FDR: false discovery rate.

Supplementary Dataset 2 (Supplementary Dataset 2.xlsx)

List of 442 differentially expressed genes (FDR ≤ 0.05; $log_2FC \geq \pm 1$ or $log_2FC \leq - 1$) exclusively in the 07-06h-ZH group versus normoxia. Given are the official gene name, ensembl number (Identifier), gene description, the log_2FC over normoxic controls, FDR and p-value. FC: fold change; FDR: false discovery rate.

Supplementary Dataset 3 (Supplementary Dataset 3.xlsx)

Genes in the top 4 sets identified in the gene set enrichment analysis (GSEA) of 07-06h-ZH compared to normoxia with MSigDB hallmark datasets. Listed are genes contributing to the core enrichment (defined as genes most contributing to the enrichment results) of each set. Given are the rank according to enrichment, official gene name, ensembl number (Identifier), gene description, the log_2FC over normoxic controls, FDR, and p-value. Tab1: Hallmark hypoxia; Tab2: Hallmark glycolysis; Tab3: Hallmark TNFa signaling; Tab4: Hallmark coagulation. FC: fold change; FDR: false discovery rate.

Supplementary Dataset 4 (Supplementary Dataset 4.xlsx)

List of differentially expressed genes (FDR ≤ 0.05; log_2FC ≥ + 1 or log_2FC ≤ 1) in hypobaric versus normobaric 14% hypoxia. Given are the official gene name, ensembl number (Identifier), gene description, the log_2FC over normoxic controls, FDR and p-value. Tab1: 14-48h-JFJ over 14-48h-ZH; Tab2: 14-07w-JFJ over 14-11w-ZH. FC: fold change; FDR: false discovery rate.

Supplementary Dataset 5 (Supplementary Dataset 5.xlsx)

Overview of RNA-Seq datasets comparing hypoxic groups to normoxia. Unfiltered RNA-Seq datasets used for the analysis in this manuscript. The reference genome used (GRC38.p5; https://www.ncbi.nlm.nih.gov/assembly/GCF_000001635.25/) contains 25,700 genes. Given are official gene name, ensembl number (Identifier), gene description, the log_2FC over normoxic controls, FDR, and p-value for each gene. Individual tabs show values of each gene over normoxia for each hypoxic condition: Tab1: 07-06h-ZH; Tab2: 14-06h-ZH; Tab3: 14-48h-ZH; Tab4; 14-11w-ZH; Tab5: 14-48h-JFJ; Tab6: 14-07w-JFJ. FC: fold change; FDR: false discovery rate.
